# From Caudal Regression to Wieacker-Wolff Syndrome: The Decisive Role of Postmortem Examination and Exome Sequencing in Revising a Prenatal Diagnosis

**DOI:** 10.7759/cureus.95639

**Published:** 2025-10-29

**Authors:** Marie Nicoletti, Camille Gilmard, Aude Tessier, Patricia Steenhaut

**Affiliations:** 1 Department of Obstetrics, Université Catholique de Louvain, Brussels, BEL; 2 Department of Genetics, Institut de Pathologie et de Génétique, Charleroi, BEL

**Keywords:** caudal regression syndrome, fetal anomalies, gene zc4h2, prenatal diagnosis, wieacker-wolff syndrome, xq11.2 deletion

## Abstract

Prenatal detection of bilateral clubfoot should prompt a systematic evaluation for associated anomalies, particularly sacral defects and other lower-limb malformations. Arthrogryposis must also be considered in the differential diagnosis. Importantly, prenatal imaging should be complemented by postnatal assessments, which may, as in the present case, uncover a misdiagnosis with major implications for future pregnancies.

This case report illustrated how detailed postnatal investigations led to the revision of an initial diagnosis of caudal regression syndrome to the final, exceptionally rare diagnosis of Wieacker-Wolff Syndrome. Such diagnostic correction carries substantial consequences for genetic counseling.

Wieacker-Wolff syndrome, also referred to as intellectual disability, developmental delay, and contractures syndrome, is a severe neurodevelopmental disorder characterized by arthrogryposis and intellectual disability. It is inherited in an X-linked manner, either dominant or recessive, and is caused by pathogenic variants in the *ZC4H2* gene.

## Introduction

Wieacker-Wolff syndrome is a rare genetic cause of neuromuscular arthrogryposis, resulting from pathogenic variants in the *ZC4H2* gene [1]. Such variants are detected in fewer than 5% of fetal akinesia cases [2]. By 2019, only 23 families and sporadic cases had been reported with *ZC4H2* defects, comprising 19 affected female patients from 18 families and 14 affected male patients from nine families [3]. By 2021, merely six prenatal cases had been described [4]. The present report therefore represents the seventh prenatal case of Wieacker-Wolff syndrome.

The inheritance pattern of this condition is complex and may be X-linked dominant or X-linked recessive, depending on the variant type (loss-of-function versus missense) and its genomic location. The *ZC4H2* gene is predominantly expressed in the brain during development, underscoring its crucial role in fetal neurodevelopment [5].

Arthrogryposis multiplex congenita (AMC) represents a heterogeneous group of disorders characterized by multiple joint contractures present at birth, usually resulting from reduced fetal movements. The etiology is broad, including neuromuscular, neurogenic, connective tissue, and central or peripheral nervous system causes.

Some clinical features such as multiple joint contractures, limb deformities, and motor impairment may occur in both caudal regression syndrome (CRS) and neuromuscular arthrogryposis, which can complicate the prenatal differential diagnosis.

Wieacker-Wolff syndrome manifests with a broad spectrum of clinical consequences [6]. The hallmark features include arthrogryposis (multiple, permanent joint contractures restricting limb mobility) and generalized muscle weakness. This weakness may result in respiratory distress, facial ptosis, and feeding difficulties related to bulbar muscle involvement.

Skeletal anomalies are frequently observed, including camptodactyly, hip dislocation, scoliosis, kyphosis, lordosis, and talipes equinovarus (clubfoot) [7]. Distinctive facial dysmorphism is also common, typically characterized by a long and flat philtrum, low-set ears, a carp-shaped mouth, and a high-arched palate.

Intellectual disability and motor developmental delay are consistently reported, with additional neurological features such as spasticity and seizures described in some cases [8].

## Case presentation

We report the case of a 39-year-old woman, gravida 6 para 4, referred to our fetal medicine unit for suspected bilateral clubfoot. Her obstetric history included three full-term vaginal deliveries and one cesarean section at 35 weeks’ gestation for acute fetal distress in the context of intrauterine growth restriction.

The patient and her partner were of Caucasian origin and non-consanguineous. There was no personal or family history of joint contractures, neuropathy, musculoskeletal abnormalities, or intellectual disability. No history of exposure to medications, infections, trauma, or vascular insults was reported during the current or previous pregnancies.

The first-trimester ultrasound was unremarkable. Non-invasive prenatal testing for common trisomies yielded normal results. The fetus was male.

An early anomaly scan performed at 15 weeks’ gestation showed hyperextended and crossed legs, with bilateral abnormal foot alignment. No movements were observed in the lower limbs, and only limited motion of the pelvis and upper limbs was noted. These findings raised the suspicion of arthrogryposis affecting the lower extremities.

Amniocentesis was subsequently performed. The fetal conventional karyotype did not reveal any chromosomal abnormalities that could explain the phenotype. The procedure was complicated by preterm premature rupture of membranes.

**Figure 1:**
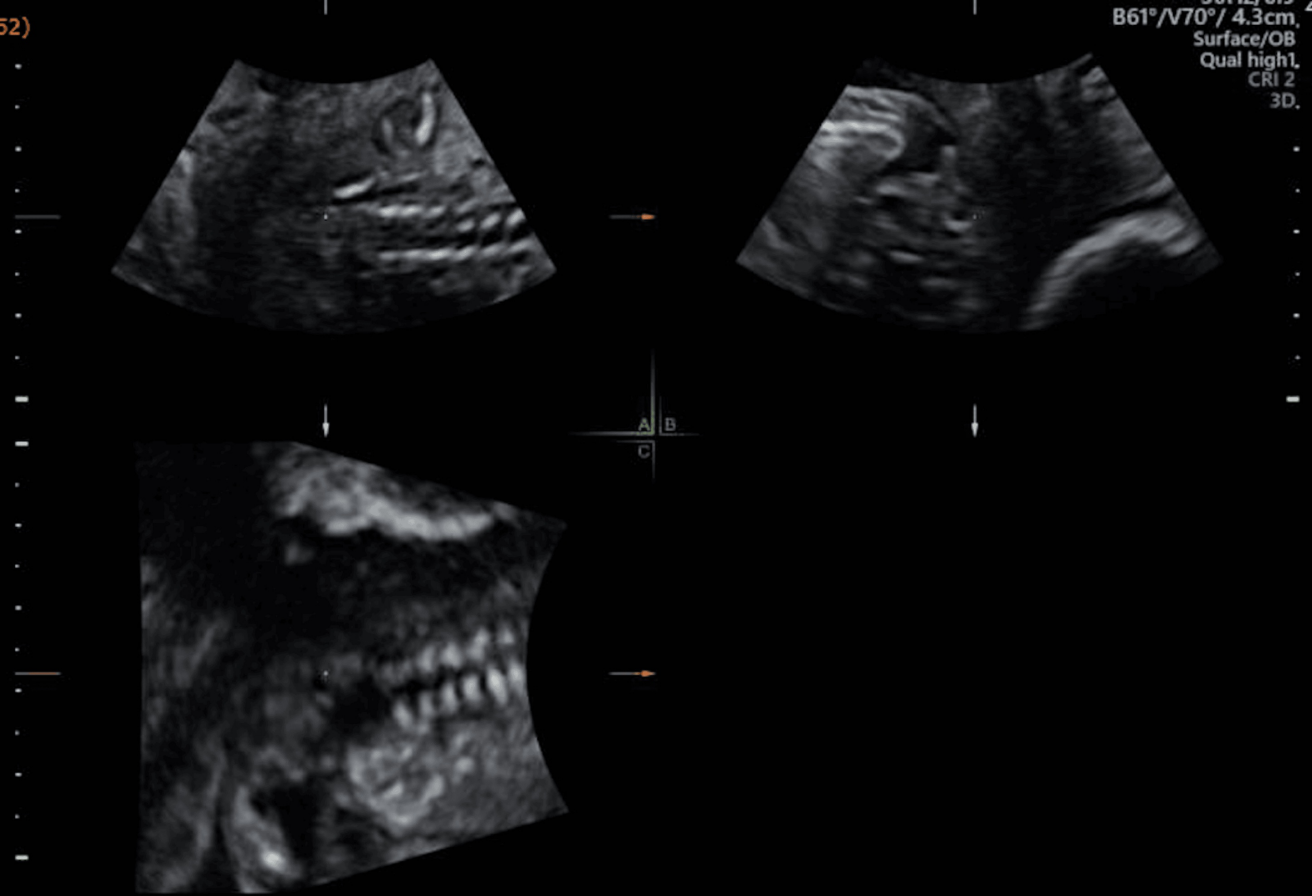
Oligohydramnios and sacral agenesis suggesting caudal regression syndrome

At 16 weeks and 4 days‘ gestation, ultrasound demonstrated oligohydramnios and sacral agenesis (Figure 1), suggesting CRS. Because of the severe oligohydramnios, reassessment of the leg and foot alignment was not feasible. Although the remainder of the fetal anatomy appeared normal, evaluation was incomplete; the gallbladder, anterior abdominal wall, and heart could not be fully assessed.

At the couple’s request, a medical termination of pregnancy was performed at 17 weeks and 4 days of gestation, in accordance with Belgian legislation. A fetal autopsy was carried out with the parents’ consent.

**Figure 2:**
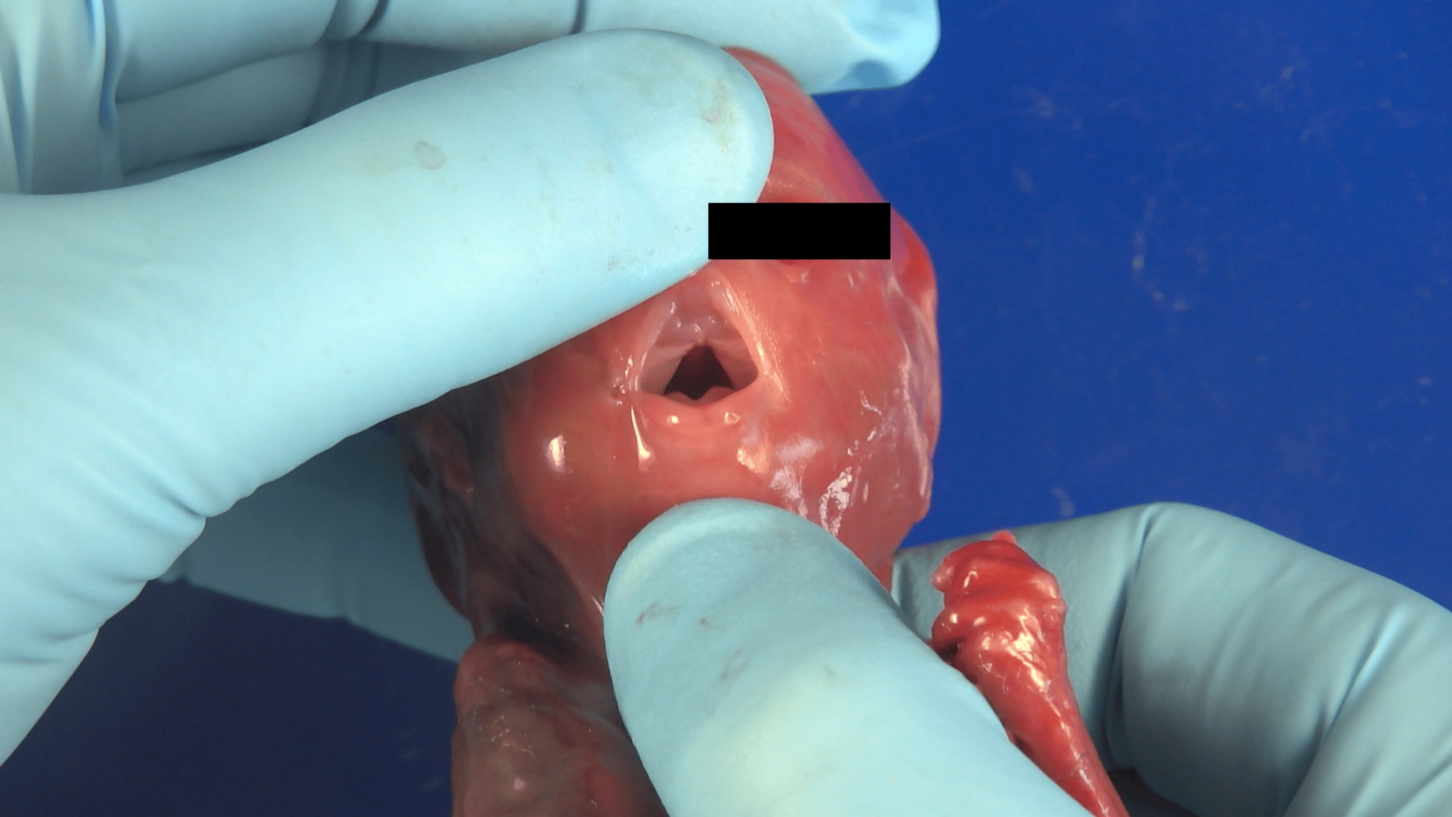
Choanae patent Open posterior nasal apertures (patent choanae) observed during autopsy. The image shows the nasal cavity and choanal openings visualized after retraction of the palate, confirming the absence of choanal atresia.

The autopsy revealed a eutrophic male fetus. Craniofacial anomalies included severe retrognathia, a cleft palate, and a small, elevated tongue, consistent with Pierre Robin sequence. The choanae were patent (Figure 2). A short, broad neck with mild edema was noted. The external genitalia were male, with intra-abdominal testes appropriate for the gestational age.

**Figure 3:**
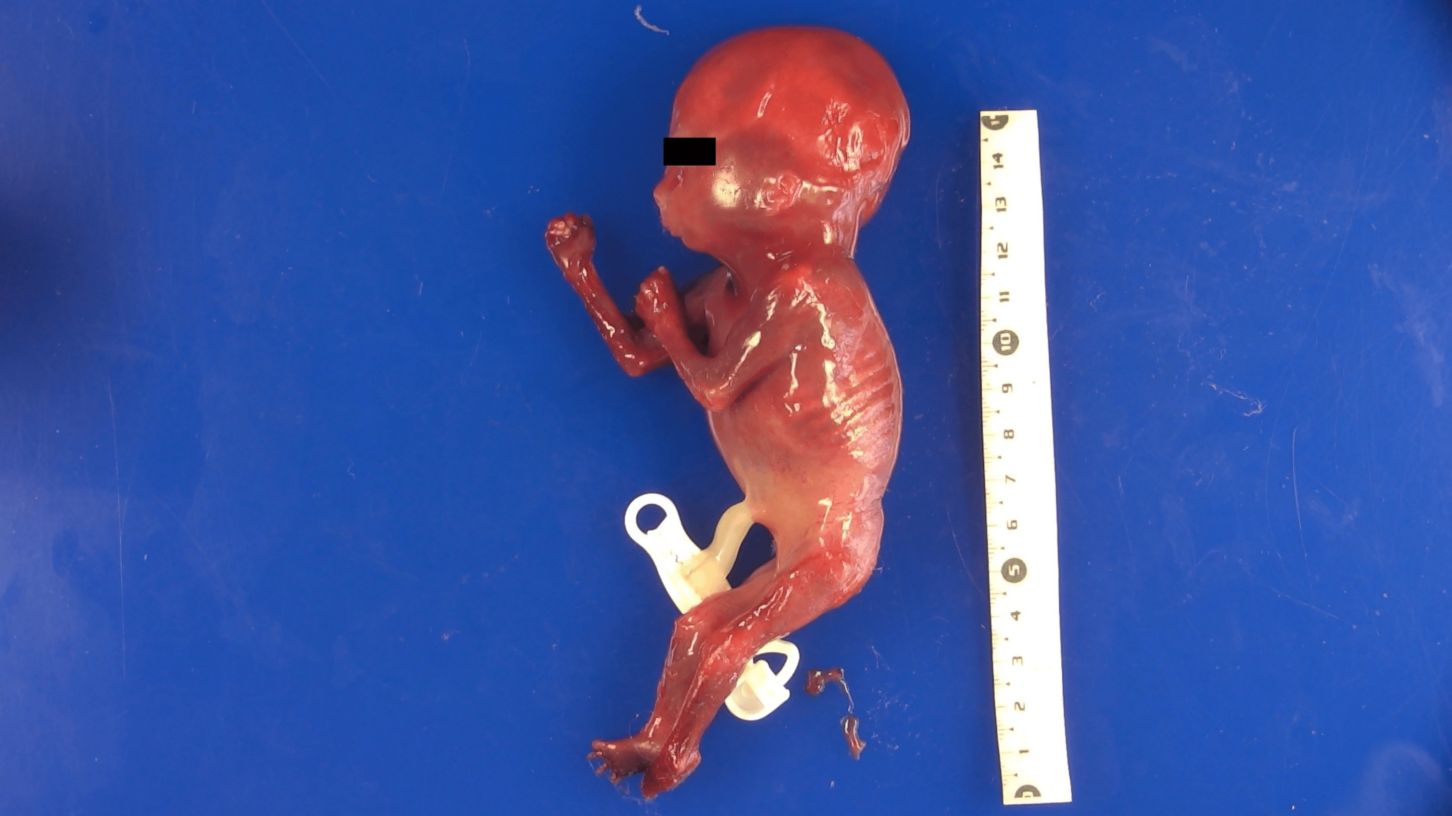
Wide gap and distal muscle atrophy External view of the fetus showing flexed thighs, hyperextended legs, abnormal foot positioning, and distal muscle atrophy. A wide gap between the first and second toes is evident, consistent with lower limb arthrogryposis and features of caudal regression syndrome.

The spine showed abnormal alignment with a kyphotic configuration but no evidence of a neural tube closure defect. All joints exhibited contractures, with fixed elbow flexion and bilateral camptodactyly. The absence of palmar flexion creases suggested impaired hand mobility. The thighs were flexed over the abdomen, the legs were hyperextended, and the feet were malpositioned. A wide gap between the first and second toes was observed, along with distal muscle atrophy (Figure 3).

**Figure 4:**
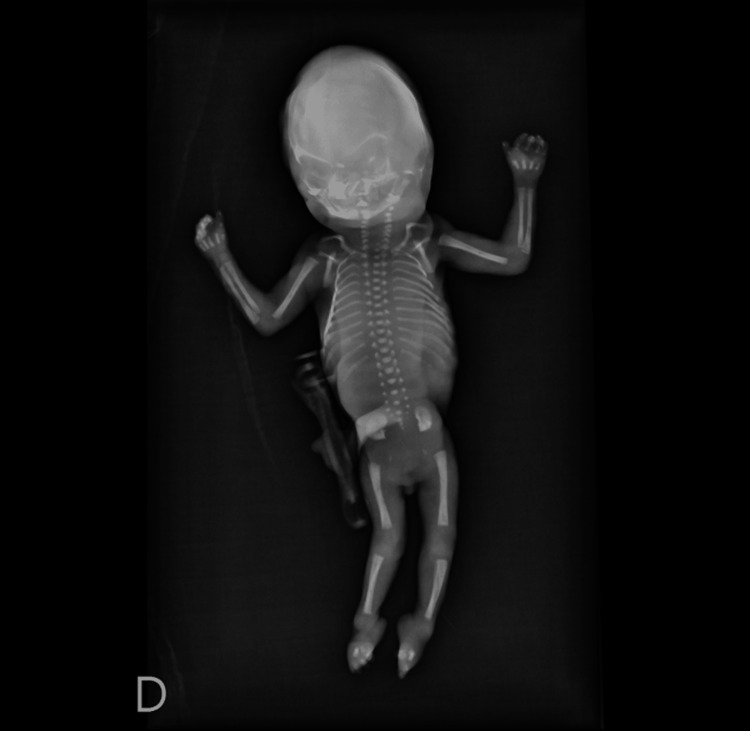
Skeletal radiographs No abnormalities, particularly no sacral defect, were present.

The visceral examination revealed pulmonary hypoplasia and a tubular-shaped stomach, consistent with impaired swallowing. Skeletal radiographs (Figure 4) showed no abnormalities, including no sacral defect.

Histological analysis of muscle tissue revealed fibers of variable size, some atrophic, with probable excess fibrosis, but without fatty degeneration or glycogen accumulation. Neuropathological examination showed no abnormalities of the spinal cord. Placental examination revealed no significant macroscopic or histological findings.

The overall autopsy findings were consistent with arthrogryposis of neuromuscular origin (Table 1).

**Table 1:** Comparison between prenatal ultrasound and postmortem findings Summary of key prenatal ultrasound findings compared with postmortem examination results in the present case. Prenatal imaging suggested caudal regression syndrome, whereas autopsy and genetic analyses confirmed Wieacker-Wolff syndrome due to a de novo ZC4H2 variant.

Feature	Prenatal ultrasound findings	Postmortem findings
Limb movement	Absent in lower limbs; limited in upper limbs	Generalized joint contractures and muscle atrophy
Limb posture	Hyperextended legs; crossed position	Fixed flexion of thighs and hyperextended legs
Feet	Bilateral abnormal alignment (suspected clubfoot)	Talipes deformity and widened first–second toe gap
Spine	Sacral agenesis suspected	Kyphotic alignment; no neural tube defect
Craniofacial	Not evaluated prenatally	Pierre Robin sequence (retrognathia, cleft palate, glossoptosis)
Thoraco-abdominal organs	Poorly visualized (oligohydramnios)	Pulmonary hypoplasia; tubular stomach
Genetic testing	Normal karyotype and SNP-array	ZC4H2 variant c.592C>T, p.(Arg198Trp) identified

These results were discussed with the couple during a post-termination genetic counseling session. To further investigate the etiology, trio-based exome sequencing was proposed to identify potential monogenic causes of arthrogryposis.

Trio-based exome sequencing was performed using the KAPA HyperExome V2 capture kit (Roche) and Illumina sequencing, as part of a validated next-generation sequencing protocol as previously described by Meunier et al. [9]. The mean coverage depth was approximately 140×, and data were analyzed with in-house bioinformatics pipelines (NGS-Pipe and Variant Explorer). Variants were aligned to the GRCh38 reference genome and filtered according to inheritance model, population frequency (gnomAD v4.1.0), predicted impact, and Human Phenotype Ontology (HPO) terms [10] (Pierre Robin sequence, AMC, generalized amyotrophy). Variant interpretation followed the ACMG guidelines [11], and the *ZC4H2* variant (c.592C>T, p.Arg198Trp) was validated by Sanger sequencing in the proband and confirmed to be de novo.

Analysis identified a hemizygous variant in *ZC4H2* (NM_018684.4, GRCh38): c.592C>T, p.(Arg198Trp). This variant is classified as likely pathogenic/pathogenic in the ClinVar (432364) and has been previously reported in a heterozygous female with AMC and intellectual disability.

**Figure 5:**
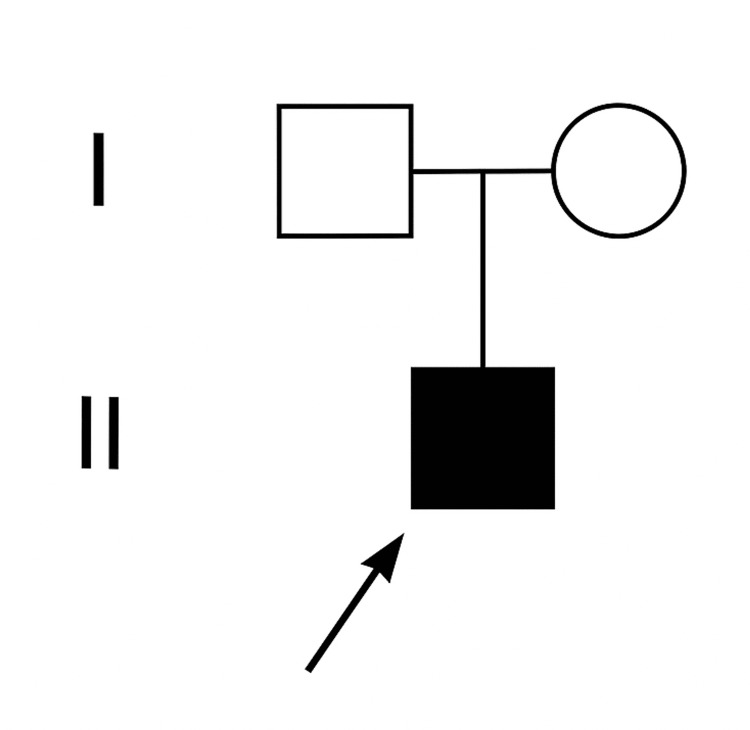
Pedigree of the family showing the affected male proband with a de novo hemizygous ZC4H2 variant. Both parents tested negative for the variant

Pathogenic *ZC4H2* variants are associated with Wieacker-Wolff syndrome, an X-linked disorder with dominant or recessive inheritance. Given the phenotypic concordance, the *ZC4H2* variant (NM_018684.4, GRCh38: c.592C>T, p.(Arg198Trp)) was deemed causal. As it was absent in both parents, a de novo origin was most likely, although germline mosaicism could not be excluded (Figure 5).

## Discussion

This case highlights the importance of complementing antenatal investigations with post-mortem examinations whenever feasible. Although prenatal ultrasound enables the detection of a wide range of anomalies, it also has intrinsic limitations. In this case, severe oligohydramnios compromised image quality, leading to the erroneous suspicion of sacral agenesis. When considered alongside bilateral clubfoot, these findings resulted in a presumptive-but incorrect-diagnosis of CRS. This underscores the pivotal role of postnatal fetal assessments.

Comparable diagnostic difficulties have been reported in previously published cases of Wieacker-Wolff syndrome and other *ZC4H2*-related disorders. Wieacker and Wolff first described the syndrome in 1985 in males presenting with arthrogryposis, muscle atrophy, and oculomotor apraxia [6]. Later molecular studies, such as those by Hirata et al. (2013) [5] and Frints et al. (2019) [3], identified pathogenic or de novo variants in *ZC4H2*, confirming the X-linked nature of the condition and the variable phenotype observed in both sexes.

More recently, Deneufbourg et al. (2021) [4] described a prenatal case with congenital arthrogryposis multiplex and a partial deletion of *ZC4H2*, sharing several features with our observation-including lower-limb contractures, fetal akinesia, and normal cranial anatomy. As in our report, postmortem examination and molecular analysis were essential to refine the diagnosis, underscoring how *ZC4H2* anomalies may mimic caudal regression or other neuromuscular syndromes during prenatal imaging. Similarly, Wongkittichote et al. (2023) [2] and Godfrey et al. (2018) [1] expanded the genotypic and phenotypic spectrum of these disorders, illustrating their heterogeneity and the diagnostic complexity they present.

From a differential diagnosis perspective, fetal arthrogryposis may result from a wide spectrum of disorders. Neuromuscular causes include spinal muscular atrophy, fetal myopathies, and congenital myasthenic syndromes, whereas neurogenic causes encompass brain malformations, anterior horn cell disorders, and spinal dysraphism. Extraneuromuscular etiologies such as connective tissue disorders (e.g., Larsen syndrome), chromosomal anomalies, or maternal factors (e.g., myasthenia gravis, uterine constraint, or vascular insults) should also be considered. The identification of* ZC4H2* variants among these conditions emphasizes the need for comprehensive genetic evaluation in all unexplained cases of fetal akinesia or multiple joint contractures.

Both skeletal radiography and autopsy in our case provided additional evidence that justified the use of trio exome sequencing. As in the reports by Frints et al. (2019) [3] and Deneufbourg et al. (2021) [4], the integration of genomic analysis was essential to confirm the diagnosis and to distinguish *ZC4H2*-related arthrogryposis from other etiologies.

The exome sequencing achieved a mean coverage depth of approximately 140×, providing sufficient quality for reliable variant detection. All candidate variants were confirmed by Sanger sequencing in the proband and both parents, validating the de novo origin of the *ZC4H2* variant. Variant classification followed the ACMG recommendations [11] and supported a pathogenic status (class 5), consistent with the criteria typically applied for de novo missense variants affecting highly conserved residues (PS2, PM1, PM2, PM5, PP2, PP5). These methodological steps ensured the robustness of the molecular diagnosis and strengthened the interpretation of the genotype-phenotype correlation in this case.

Exome analysis ultimately identified an exceptionally rare syndrome which, in addition to contributing to the medical literature, provided the basis for precise genetic counseling in anticipation of future pregnancies.

From a genetic counseling standpoint, our findings are consistent with prior reports emphasizing that most pathogenic *ZC4H2* variants occur de novo [3-5], resulting in a low recurrence risk, although germline mosaicism cannot be fully excluded.

Given the severe phenotype observed in male fetuses, serial ultrasound monitoring may enable prenatal recognition. However, when ultrasound findings are unremarkable, invasive testing can be offered for reassurance. Chorionic villus sampling at approximately 12 weeks’ gestation or amniocentesis at around 16 weeks’ gestation would allow definitive exclusion of the diagnosis.

Overall, by comparing our findings with those previously published, this case broadens the prenatal spectrum of Wieacker-Wolff syndrome and underlines the decisive contribution of autopsy and exome sequencing in resolving ambiguous prenatal diagnoses. The addition of detailed technical validation and variant classification ensures the reliability of the molecular findings, while the inclusion of differential diagnoses and summarized literature data provides valuable clinical guidance for future prenatal and postnatal cases (Table 2).

**Table 2:** Reported pathogenic variants in the ZC4H2 gene and their inheritance pattern Summary of previously reported pathogenic ZC4H2 variants, associated phenotypes, and inheritance patterns. AMC = arthrogryposis multiplex congenita.

Variant (NM_018684.4)	Protein change	Sex	Inheritance pattern	Reported phenotype	Reference
c.70C>T	p.(Arg24Trp)	Female	X-linked dominant	AMC, intellectual disability, facial dysmorphism	Hirata et al., 2013 [5]
c.592C>T	p.(Arg198Trp)	Male	De novo, X-linked recessive	AMC, Pierre Robin sequence, muscle atrophy	Present case
c.310G>A	p.(Gly104Ser)	Male	X-linked recessive	Arthrogryposis, spasticity, mild cognitive delay	Frints et al., 2019 [3]
c.553C>T	p.(Arg185Trp)	Female	X-linked dominant	AMC, intellectual disability	Deneufbourg et al., 2021 [4]
c.126_127del	p.(Glu43Glyfs*12)	Male	De novo	Severe AMC, hypotonia	Godfrey et al., 2018 [1]
c.745A>G	p.(Asn249Ser)	Female	X-linked dominant	Mild AMC, learning difficulties	Wongkittichote et al., 2023

## Conclusions

This case highlights the importance of a multidisciplinary and integrated approach in the prenatal diagnosis of congenital anomalies. While ultrasound and imaging techniques remain central, their limitations, particularly in the context of severe oligohydramnios, must be acknowledged. Fetal autopsy and exome sequencing enabled the revision of an initial suspicion of caudal regression syndrome to the precise diagnosis of Wieacker-Wolff syndrome, an exceptionally rare genetic disorder. This diagnostic clarification not only contributed to the medical literature but also had a direct impact on genetic counseling and the management of future pregnancies for this family. More broadly, this case underscores the need to complement morphological assessments with genetic investigations in order to improve diagnostic accuracy and optimize parental care.
